# Varying levels of natural light intensity affect the phyto-biochemical compounds, antioxidant indices and genes involved in the monoterpene biosynthetic pathway of *Origanum majorana* L.

**DOI:** 10.1186/s12870-024-05739-5

**Published:** 2024-10-28

**Authors:** Zahra Hashemifar, Forough Sanjarian, Hassanali Naghdi Badi, Ali Mehrafarin

**Affiliations:** 1 Department of Plant Bio-Products, Institute of Agricultural Biotechnology (IAB), National Institute of Genetic Engineering and Biotechnology (NIGEB), Tehran, 1497716316 Iran; 2 Department of Agronomy and Plant Breeding, Faculty of Agriculture, Shahed University, Tehran, 3319118651 Iran; 3 Medicinal Plants Research Center, Shahed University, Tehran, 3319118651 Iran

**Keywords:** Abiotic stress, Lamiaceae, Majoram, Shading, Sustainable agriculture, Thymol

## Abstract

**Background:**

Light is a critical environmental factor in plants, encompassing two vital aspects: intensity and quality. To assess the influence of different light intensities on *Origanum majorana* L., pots containing the herb were subjected to four levels of light intensity: 20, 50, 70, and 100% natural light. After a 60-day treatment period, the plants were evaluated for metabolite production, including total sugar content, protein, dry weight, antioxidant indices, expression of monoterpenes biosynthesis genes, and essential oil compounds. The experimental design followed a randomized complete blocks format, and statistical analysis of variance was conducted.

**Results:**

The results indicated a correlation between increased light intensity and elevated total sugar and protein content, which contributed to improved plant dry weight. The highest levels of hydrogen peroxide and malondialdehyde (MDA) were observed under 100% light intensity. Catalase and superoxide dismutase enzymes exhibited increased activity, with a 4.23-fold and 2.14-fold increase, respectively, under full light. In contrast, peroxidase and polyphenol oxidase enzyme activities decreased by 3.29-fold and 3.24-fold, respectively. As light intensity increases, the expression level of the *1-deoxy-D-xylulose 5-phosphate reductoisomerase (DXR)* gene increases. However, beyond a light intensity of 70%, the *DXR* gene expression level decreased. Furthermore, the expression levels of the cytochrome P450 genes *CYP71D178* and *CYP71D179* exhibited an increasing trend in response to elevated light intensity. Essential oil content increased from 0.02 to 0.5% until reaching 70% light intensity. However, with further increases in light intensity, the essential oil content decreased by 54 to 0.23%.

**Conclusions:**

These findings emphasize the importance of balancing plant growth promotion and stress management under different light conditions. The research suggests that sweet marjoram plants thrive best in unshaded open spaces, resulting in maximum biomass. However, essential oil production decreases under the same conditions. For farmers in areas with an average light intensity of approximately 1700 µmol m^−2^s^−1^, it is recommended to cultivate sweet marjoram in shade-free fields to optimize biomass and essential oil production. Towards the end of the growth cycle, it is advisable to use shades that allow 70% of light to pass through. The specific duration of shade implementation can be further explored in future research.

**Supplementary Information:**

The online version contains supplementary material available at 10.1186/s12870-024-05739-5.

## Introduction

Plants possessing medicinal properties offer a vast bioresource that provides solutions for traditional medicine, modern pharmaceuticals, nutraceuticals, dietary supplements, traditional remedies, essential components for pharmaceutical synthesis and intermediates for chemical compounds. Additionally, aromatic plants serve as important sources of fragrances, flavors, cosmeceuticals, health-enhancing beverages, and essential chemical terpenes [1, 2].

Medicinal plants, as defined by the World Health Organization (WHO), are species that occur naturally or are intentionally cultivated and contain various bioactive compounds capable of preventing, alleviating, or treating diseases. These bioactive compounds are produced through primary and secondary metabolic pathways and are called secondary metabolites. The plant kingdom boasts a wide range of secondary metabolites, many of which possess significant biological activity [3].

*Origanum majorana* L. (OM), commonly referred to as Sweet Marjoram, is a perennial herb indigenous to the Mediterranean region and belongs to the Lamiaceae family. This herb has a longstanding history of traditional use across various cultures, particularly in the realm of traditional medicine [3]. OM contains many bioactive compounds, such as essential oils, flavonoids, and phenolic acids. The key constituents identified in the essential oil of OM include terpinen-4-ol, γ-terpinene, and sabinene, which play a vital role in its therapeutic properties. Recognized for its anti-inflammatory, antimicrobial, and antioxidant attributes [3], OM has been extensively employed in traditional herbal medicine to address various conditions, including digestive disorders, respiratory ailments, and menstrual cramps [4].

Plants often face fluctuations in light intensity, leading to either an excess or deficiency of light. These fluctuations, referred to as light stress, significantly affect the agronomic characteristics of plants. This impact arises from the disruption of essential physiological processes, including photosynthesis, the functioning of antioxidant systems, and the plant's capacity to assimilate atmospheric carbon and nitrogen [5].

Light stress-induced disturbances in plant physiology frequently result in oxidative stress, characterized by the excessive buildup of reactive oxygen species (ROS) within plant cells. The accumulation of ROS, such as superoxide radicals, hydrogen peroxide, and hydroxyl radicals, can trigger detrimental oxidative reactions. Consequently, this oxidative stress has negative effects on numerous cellular components, including lipids, proteins, and nucleic acids, thereby compromising the overall well-being and vitality of the plant [6].

To counteract the detrimental effects of oxidative stress, plants have developed a robust defense system that involves antioxidant enzymes. Enzymes, such as superoxide dismutase (SOD), catalase (CAT), and peroxidases, play a crucial role in neutralizing reactive oxygen species (ROS) and preventing cellular damage. They facilitate the conversion of harmful ROS into less reactive or non-toxic forms, thereby preserving the integrity of essential cellular structures. In addition to enzymatic defenses, plants also employ non-enzymatic antioxidants, primarily phenolic compounds and flavonoids. These secondary metabolites serve as essential components of the plant's antioxidant machinery. Phenolic compounds and flavonoids possess antioxidant properties by scavenging ROS and alleviating oxidative stress [6]. Moreover, these compounds exhibit photo-protective effects and can chelate iron, which is significant in preventing iron-induced damage to lipids and proteins in the cellular membrane. The presence and concentration of phenols and flavonoids in plant tissues can greatly influence the plant's resilience to fluctuations in light intensity and the resulting oxidative stress. By effectively neutralizing ROS and counteracting the damage they cause, phenols and flavonoids contribute to the plant's capacity to adapt and thrive in different light conditions [6].

Essential oils also referred to as volatile or ethereal oils, are aromatic oily substances extracted from various plant sources, including flowers, seeds, leaves, twigs, roots, and fruits. Numerous plants contain these oils, which predominantly consist of diverse secondary metabolites, including terpenoids. These compounds are typically stored within different secretory glands and trichomes present in plants [7, 8].

The impact of light intensity on essential oil production in the Lamiaceae family, comprising aromatic herbs like thyme, mint, lavender, rosemary, and basil, has been extensively researched. Research has demonstrated that light intensity plays a critical role in the biosynthesis of essential oils in these plants. Maintaining an optimal balance of light intensity is essential for the synthesis of essential oils. Both insufficient and excessive light can have negatively impact on their production. Insufficient light may restrict photosynthesis and reduce the availability of precursor molecules for essential oil biosynthesis. Conversely, excessive light can result in photo-inhibition and oxidative stress, which can impede the production of essential oils. Consequently, it is essential to regulate and optimize light intensity to maximize the yield of essential oils in Lamiaceae plants [9, 10].

*Hyptis suaveolens* (Family Lamiaceae) grew the fastest in full sunlight. The decrease in light intensity made this plant not flower. In this situation, there was an increase in the number of essential oil compounds [11]. The accumulation of arbutin in *Origanum majorana* L. was found to be influenced by light intensity and wavelength. Specifically, the highest amount of photosynthetic pigments was produced under the lowest light intensity and green light. Furthermore, the maximum dry biomass was observed under light intensity. The accumulation of essential oil was observed to be highest at light intensities exceeding 130 [12]. The variation in light intensity led to a corresponding alteration in the quantity of essential oil present in *Rosmarinus officinalis* L. [13].

Given the medicinal importance of the essential oil from the OM plant, which contains compounds such as thymol, it is essential to cultivate it at both micro and macro levels. Small-scale farmers in sustainable cultivation and mixed cultivation systems should know what the minimum light requirement of this plant is and can this plant be cultivated under the shade of plants. In addition, in greenhouse cultivation, knowing the optimal light intensity for OM helps to achieve higher quality of products and also save energy.

In this study on OM, we conducted experiments exposing the plants to varying intensities of light. Subsequently, we compared them based on their phytochemical traits, metabolites, expression of genes related to their progression pathway and enzymatic and non-enzymatic antioxidant activity.

## Material and methods

### Plant materials and experimental site

The pots of OM were obtained from the Institute of Medicinal Plant of the Academic Center for Education, Culture, and Research (ACECR) situated at coordinates 35.90 N and 50.88 E, at an altitude of approximately 1475 m above sea level. These pots were placed in a greenhouse maintained at a temperature of 25 ºC, with 60% humidity and a photoperiod of 16 h of light followed by eight h of darkness, where they were nurtured for six months. Once the plants were well-established, they were transitioned from the greenhouse to outdoor conditions and exposed to varying light intensities for two months. To achieve varying levels of light intensity, polyethylene shade nets of different thicknesses were employed, resulting in light intensities of 20, 50, and 70%, respectively. Light intensity beneath each shade was measured using a lux meter. (INS DX-200, INS Enterprise Co., Ltd., Taiwan). This experiment was conducted using a randomized block design. The shading materials were positioned at a height of 80 cm above the plant surfaces. Concurrently, a set of *Origanum majorana* L. were placed outdoors without covering as control samples. Throughout the growth period, the radiation intensity ranged from 1100 to 2270 μmol m^−2^ s^−1^ (typical for full sunlight on a sunny day) (mean = 1682 μmol m^−2^ s^−1^).

The air temperature in the shaded area was consistent with the temperature outside due to the natural wind and air circulation, resulting in a uniform temperature. At 9, 12, and 18 h, the average air temperatures were recorded as 27.42, 28.85, and 20.29 degrees Celsius, respectively. After 60 days, the shoot was harvested and transferred to the laboratory at the National Institute of Genetic Engineering and Biotechnology (NIGEB), using liquid nitrogen. The plant samples were stored in a freezer at a temperature of -70 ºC until the time of measurement.

### Protein and total sugar content

The samples were frozen in liquid nitrogen, crushed, and added to a sodium phosphate buffer (50 mM, pH = 7). Subsequently, centrifugation was carried out. The supernatant obtained was used to measure the total protein concentration using the Bradford method. BSA was used as the standard reference [14]. This extract was also utilized for enzyme measurements. The concentration of total sugar content was determined calorimetrically using the anthrone method [15].

### Antioxidant enzymes and components

200 mg shoot samples were ground in 2 ml phosphate buffer solution and centrifuged at 4 °C for 20 min at 10,000 g. The supernatant was tested for total protein concentration and enzyme activities using the Bradford method with BSA as a standard [14].

#### Superoxide dismutase (SOD) enzyme

The reaction solution comprised a phosphate buffer, 13 mM methionine, 75 μM NBT, 0.200 mM EDTA, 20 μM riboflavin, and 180 μl of protein extract. Subsequently, this reaction solution was subjected to light radiation for 20 min, following which the absorbance was measured at 560 nm. The quantification of SOD activity is expressed as U mg^−1^ of protein [16].

#### Catalase (CAT) enzyme

The 0.1 mL of leaf protein extract was combined with 100 µL of hydrogen peroxide (H_2_O_2_) and 2800 µL of phosphate buffer. The decrease in optical density (OD) at 240 nm was continuously measured to monitor the decomposition of H_2_O_2_. Absorbance measurements were taken at room temperature (20 °C) using a spectrophotometer (Specord 50, Analytik Jena, Germany) at 10-s intervals. In the control solution, the H_2_O_2_ was substituted with buffer, and the enzyme extract was also included [17].

#### Peroxidase (POD) enzyme

A reaction mixture with a total volume of 3 mL was prepared, consisting of 2.7 mL of phosphate buffer, 0.1 mL of H_2_O_2_ (0.5%), 0.1 mL of Guaiacol (80 mM), and 0.1 mL of protein extract. The absorbance of this reaction mixture was monitored at 420 nm with a spectrophotometer at 10-s intervals for a total measurement duration of 3 min, all at a temperature of 20 °C. A blank was utilized for reference, in which the enzyme extract from the shoots was substituted with a buffer solution while maintaining the other components of the mixture [18].

#### Polyphenol oxidase (PPO) enzyme

The reaction mixture comprised 0.2 ml of pyrogallol (20 mM), 2.5 ml of 50 mM phosphate buffer, and 50 μl of protein extract. The enzyme's activity was measured at 430 nm. PPO activity was expressed as U mg^−1^ of protein [19].

#### Total phenol content (TPC)

0.5 mg of plant tissues were immersed in 4 ml of absolute methanol at 37 °C for 30 min followed by centrifugation for 5 min, at 10,000 g. The obtained supernatant was utilized for quantifying the total phenol content (TPC) and total flavonoid content (TFC).

A solution was prepared using the Folin–Ciocalteu method. It involved combining 180 µl of the methanolic extract with 10 µl of distilled water and 2400 µl of Folin-Ciocalteu phenol reagent (10%).The mixture was allowed to stand at room temperature for 5 min. Subsequently, 500 µl of 20% sodium carbonate was added, and the solution was kept in complete darkness for 120 min. The absorbance was then measured at 740 nm [20].

#### Total flavonoid content (TFC)

A solution was prepared by combining 2 ml of a Methanolic extract with 100 μl of 10% aluminum chloride, 100 μl of 1M potassium acetate, and 2.8 ml of distilled water. The resulting mixture was then kept in a dark environment for 30 min, and the absorbance was measured at 415 nm. A standard curve was generated using quercetin as the reference compound [21].

#### Anthocyanin content

A methanolic extract was prepared from the shoot material using a 99:1 ratio of methanol to HCl. The extract was stored in complete darkness at 4 °C for 24 h. Following this, centrifugation was performed at 4000 rpm for 10 min. To remove any residual chlorophyll from the solution, 100 ml of chloroform was added to the supernatant. The lower phase of the solution was used to determine the concentration of anthocyanins, with absorbance readings taken at 530 nm [22].

### H_2_O_2_ content

To determine the H_2_O_2_ content, 0.2 g of plant powder was mixed with 2 ml of 0.1% (w/v) trichloroacetic acid (TCA) while maintaining it on ice. Subsequently, the solution was centrifuged at 12,000 rpm for 15 min at 4 °C. Afterward, 0.5 ml of the supernatant was combined with 0.5 ml of 0.1 mM potassium phosphate buffer at pH = 7 and 1 ml of potassium iodide (KI). The optical density (OD) of the reaction was measured at a wavelength of 390 nm [23]. The concentration of hydrogen peroxide in the plant tissue was determined using a standard curve.

### Malondialdehyde (MDA) content

To 200 mg of plant powder, 2 ml of 0.1% trichloroacetic acid (TCA) was added and centrifuged at 6000 rpm for 10 min. Subsequently, 1 ml of the supernatant was transferred to a new tube, and 4 ml of 20% TCA containing 0.5% Thiobarbituric acid (TBA) was added. The reaction mixture was then heated in a water bath at 95°C for 30 min. Afterward, the tubes were promptly placed in an ice bath. The samples were later subjected to centrifugation at 8000 rpm for 15 min. The OD was measured at wavelengths of 532 nm (absorption of the MDA-TBA complex) and 600 nm (absorption of other non-specific pigments). The extinction coefficient used for calculations was 155 mM^−1^cm^−1^ [24].

### Essential oils content and composition

The aerial parts of the plant were collected, dried in a shaded area for 2 weeks, ground, and then subjected to essential oil (EO) extraction using specialized equipment. The composition of the essential oils was assessed using a Gas Chromatograph (GC) and Gas Chromatograph-Mass Spectrometer (GC–MS).

Essential oils were extracted from the dried aerial parts through a conventional hydro-distillation. The extraction process was carried out for 3 h. The hydro-distillation procedure used a Clevenger-type apparatus, which included a 1,000 ml steam generator flask, a distillation flask, a condenser, and a receiving vessel. Subsequently, the extracted essential oils were stored at -20 °C for further analysis.

GC Analysis: An Agilent 6890 GC gas chromatograph equipped with a flame ionization detector and a DB-5 capillary column (30m × 0.25 mm, film thickness 0.25μm, type BPX5) was utilized. Helium gas (0.5 mlmin^−1^) was used as the carrier gas. The temperature program for the oven was set to 50 °C for 5 min, followed by a gradual increase to 240 °C at a rate of 3°Cmin^−1^, then a rapid increase at 15°Cmin^−1^, and finally held constant at 300 °C for 3 min.

GC/MS Analysis: An Agilent 5973 MS GC/MS system was employed with an ionization voltage of 70 electron volts, using the electron impact ionization (EI) method and maintaining the ionization source at a temperature of 220 °C.

A homologous series of n-alkanes was injected under the same conditions as the EO sample. Calculated Kovats retention indices (KI) were used to identify the contents of the EO by matching the software with the Wiley 7 N library. The retention indices and MS fragmentation patterns were then compared directly with those of standard compounds [25, 26].

### Genes expression

The expression levels of three genes—*DXR, CYP71D178,* and *CYP71D179*- associated with the monoterpenes biosynthesis pathway (MEP), were evaluated using real-time polymerase chain reaction (real-time PCR). Total RNA was extracted from the shoots of OM using an RNX-plus kit (EX6101, Sinaclon, Iran) following the instructions provided by the manufacturer. Then, cDNA synthesis was conducted using an Addbio kit (Korea). The primer annealing temperatures were optimized using gradient PCR. Subsequently, a semi-quantitative RT-PCR was conducted. For the qRT-PCR analysis, we utilized the Amplicon mastermix (ID 5000850–1250) and the Mic q-PCR cycler from Bio Molecular System. The *GAPDH* gene was utilized as a control in the experiment. The primer specifications are provided in Table 1 [27].

**Table 1:** Characteristics of primers designed for *Origanum majorana* L

Genes		primers sequence (5ʹ-3ʹ)	Optimized annealing temperature (ºC)
*DXR*	Forward Reverse	GAGGTGCTTTCAGGGATTTG AGATTGGGGATGGATAACGAT	54
*CYP71D178*	Forward Reverse	CAAGGAATGACTGCTGCTGAC TTGGATTGTGGATTGTTGGAACC	58
*CYP71D179*	Forward Reverse	CGTGGCTTCTCAACCTTCTC CGCTCTTCTTCACCCTATGC	58
*GAPDH*	Forward	TCACTGACAAGGACAAGGCTG	60
	Reverse	CTGGCTTCGCAAGTCTAACAG	

### Statistical Analyses

This study was conducted in field conditions, outside the greenhouse, using a randomized complete block design with three replications. Data normalization and analysis were performed using SAS 9.4 software. Duncan's multiple range test was utilized to compare the means. PCA analysis was carried out using SPSS 16 software. Gene expression analysis was conducted using REST 2009 software.

## Results

### Protein content and growth indicators

The protein content varied significantly among all light treatments. Specifically, in the full light treatment, the protein content was twice as high as in the 20% light treatment (Table 2, S1). As light intensity increased, both the dry weight percentage and sugar content exhibited significant increases of 23.8 and 22.36%, respectively, when compared to the lowest light intensity (Table 2, S1). Conversely, there was no significant difference in the dry weight percentage among light intensities of 20, 50, and 70%. The highest dry weight percentage was observed in the full light treatment, reaching 51.44% (Table 2, S1).

**Table 2:** The effect of different intensities of natural light on different primary metabolites in *Origanum majorana* L

**Light intensity** (%)	Protein	Dry weight (%)	Sugar content (mgl-1FW)
20	78.41^d^	41.55^b^	0.1073^b^
50	97.70^c^	42.06^b^	0.1172^b^
70	131.29^b^	46.6^b^	0.1140^b^
100	158.43^a^	51.44^a^	0.1313^a^
*p*	***	*	***

^***^p ≤ 0.001, * *p* ≤ 0.05. Values followed by the same letter in each column are not significantly different according to the Duncan test (*p* ≤ 0.05)

### Evaluation of antioxidants responses 

**Fig. 1:**
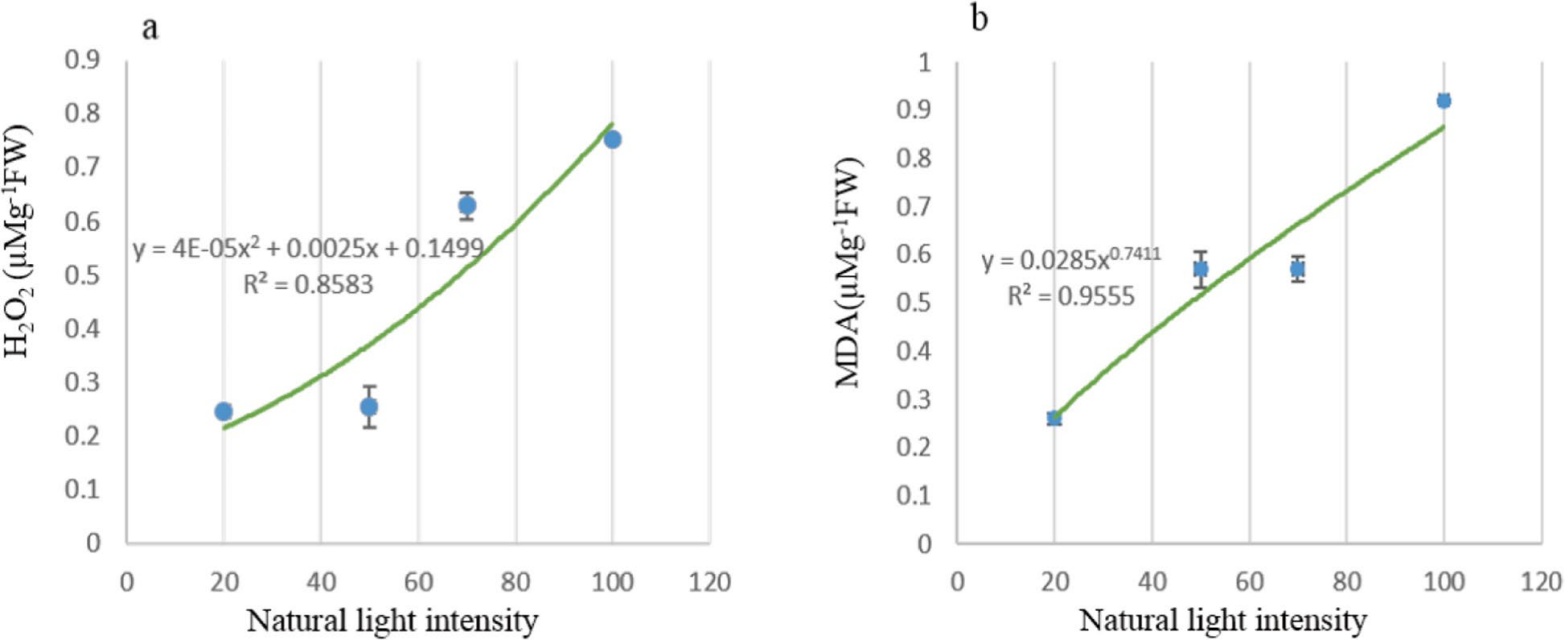
Regression diagram between changes in natural light intensity and oxidative indices (**a**. H_2_O_2_ and **b**. MDA) in aerial parts of *Origanum majorana*. R2: adjusted coefficient of determination of the regression model

H_2_O_2_ and MDA levels showed a positive correlation with light intensity. The highest levels were observed at 100% light intensity, measuring 0.92 and 0.75 µMg^−1^FW, respectively (Fig. 1, Table S1). The activity of antioxidant enzymes, specifically SOD, CAT, POD, and PPO, was measured. As light intensity increased, the activities of PPO and POD decreased significantly, with reductions of 3.74-fold and 3.29-fold, respectively, under full light conditions. In contrast, the activities of CAT and SOD increased by 4.23-fold and 2.14-fold, respectively, under the same conditions (Table 3, S2). The phenol content achieved its maximum levels of 2.2 mg g⁻^1^ FW at 20% light intensity and 2.19 mg g⁻^1^ FW at 100% light intensity. On the other hand, the lowest value was observed at 50% light intensity. Changes in light intensity did not significantly impact flavonoid content, and the variations were not statistically significant. Anthocyanin content increased with increasing light intensity, reaching its peak at 70% light intensity (0.129 µMg^−1^FW), and decreased by 16.28% under full light conditions (Table 3, S2).

**Table 3:** Changes the activity of enzymes and the concentration of antioxidant compounds in *Origanum majorana* L. shoots in response to variations in light intensity

**Light intensity** (%)	**SOD** (U mg^−1^ Protein)	**POD** (U mg^−1^ Protein)	**PPO** (U mg^−1^ Protein)	**CAT** (U mg^−1^ Protein)	**TPC (**mg.g^**−1**^ FW**)**	**TFC** **(**mg. g^**−1**^ FW**)**	**Anthocyanin (**µMg^−1^FW**)**
20	0.014^c^	38.26^a^	34.46^a^	24.27^c^	2.20^a^	0.05615^a^	0.111^b^
50	0.017^c^	49.68^a^	20.98^b^	29.34^b,c^	1.51^c^	0.05615^a^	0.114^b^
70	0.024^b^	9.62^b^	13.36^c^	61.56^b^	2^b^	0.05618^a^	0.129^a^
100	0.030^a^	11.63^b^	9.21^c^	102.65^a^	2.20^a^	0.05618^a^	0.108^b^
*p*	**	**	***	*	***	ns	*

^***^: *p* ≤ 0.001, **: *p* ≤ 0.01, *: *p* ≤ 0.05, ns: non-significant. Values followed by the same letter are not significantly different according to the Duncan test (*p* ≤ 0.05)

### Essential oils content and composition

The Marjoram essential oil content was measured under different light intensity treatments. Variance analysis of the data showed a significant difference between the light treatments (*p* < 0.01) in terms of essential oil content. The highest amount of essential oil was found at 70% light intensity (0.5%), while the lowest was at 20% (0.02%) (Table 4, S3). GC–MS analysis identified 16 compounds in OM essential oil, including 14 monoterpenes and two sesquiterpenes (Table 4). Primarily compound of the essential oil are several key compounds, including trans-sabinene hydrate, myrcene, linalool, bicyclogermacrene, terpinen-4-ol, γ-terpinene, α-terpinene, thymol, and sabinene. As the light intensity increased, the amount of thymol, sabinene, and α-terpineol increased, while the levels of myrcene and caryophyllene decreased (Table 4, S3).

**Table 4:** Variations in essential oil compounds of *Origanum majorana* L. in response to diverse levels of natural light intensity

**Components** (%)	**Natural light intensity percentage**				***p***
	**20**	**50**	**70**	**100**	
Sabinene	0.14	0.18	0.32	2.53	**
Mycenae	13.93	1.09	1.48	0.59	**
*α*-terpinene	2.4	1.65	2.55	1.18	ns
*p*-cymene	0.25	0.18	-	0.61	*
Limonene	0.19	0.16	0.14	1.44	ns
*β*-phellandrene	0.32	0.16	0.27	1.04	*
γ-terpinene	4.12	4.28	1.15	2.19	*
Linalool	0.47	0.7	1.15	4.07	*
Trans-Sabinene hydrate	50.2	50.79	49.47	60.2	*
Borneol	2.84	4.27	8.05	1.89	*
Terpinen-4-ol	2.25	2.76	3.45	3.6	ns
*α*-terpineol	0.11	0.16	0.15	3.75	**
Bornyl acetate	0.6	0.38	0.34	0.61	***
Thymol	1.36	1.72	1.54	2.67	*
Caryophyllene	4.5	2.63	1.56	1.24	*
Bicyclogermacrene	7.34	3.55	2.02	3.95	*
Essential Oil	0.02	0.32	0.5	0.23	*
Monoterpenes	79.18	67.82	70.6	86.6	*
Sesquiterpene	11.81	6.15	3.53	5.1	**

^***^p ≤ 0.001, ** *p* ≤ 0.01, *p ≤ 0.05, ^ns^ non-significant

**Fig. 2:**
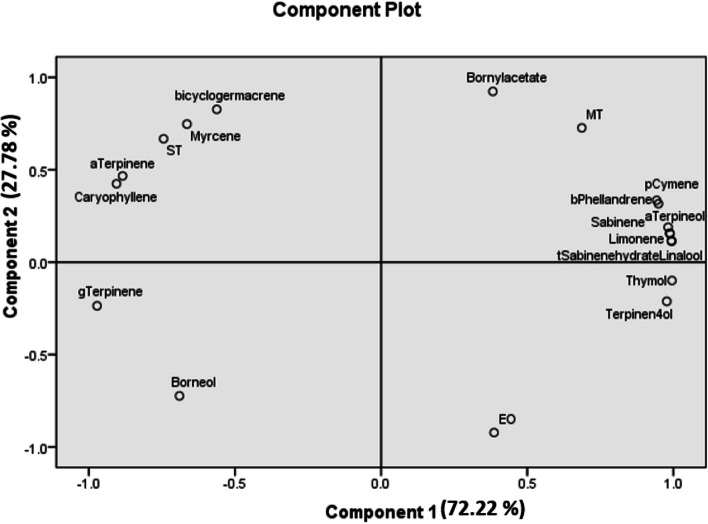
PCA component diagram of the essential oil constituents of *Origanum majorana* L. in response to variations in light condition. pCymene: *p*-cymene, bPhellandrene: *β*-phellandrene, gTerpinene: γ-terpinene, tSabinenhydrat: trans-sabinene hydrat, aTerpinene: α-terpinene, Terpinen4ol: terpinen-4-ol, aTerpineol: α-terpineol, EO: essential oil, MT: monoterpenes, ST: sesquiterpenes

Principal component analysis (PCA) was conducted to analyze the essential oil compounds. In order to identify the major components that have contribute most to total changes, we used a scree plot. Based on the scree plot, only the first two components, which accounted for 100% of the variability, were used for the analysis. The first component had a percentage of 72.22, and the second component had a percentage of 27.78. The compounds most affected by changes in light were thymol, linalool, trans-sabinene hydrate, sabinene, and *α*-terpinene (Fig. 2; Table 5, S4).

**Table 5:** Component matrix of PCA analysis of essential oil compounds in *Origanum majorana* L.

**Essential oil compounds**	**Component**	
	**1**	**2**
Sabinene	.988	.154
Myrcene	-.664	.748
*α*-Terpinene	-.884	.467
*p*-Cymene	.949	.316
Limonene	.982	.189
*β-*Phellandrene	.942	.335
γ-Terpinene	-.972	-.236
Linalool	.994	.112
Trans-sabinenehydrate	.993	.117
Borneol	-.690	-.724
Terpinen-4-ol	.977	-.212
*α*-Terpineol	.988	.157
Bornylacetate	.382	.924
Thymol	.995	-.099
Caryophyllene	-.905	.425
Bicyclogermacrene	-.562	.827
EO	.386	-.922
MT	.687	.727
ST	-.744	.668
Initial eigenvalue	13.72	5.3
Proportion (%) of variance	72.22	27.78
Cumulative of variance (%)	72.22	100

### Genes expression and molecular analysis

**Fig. 3:**
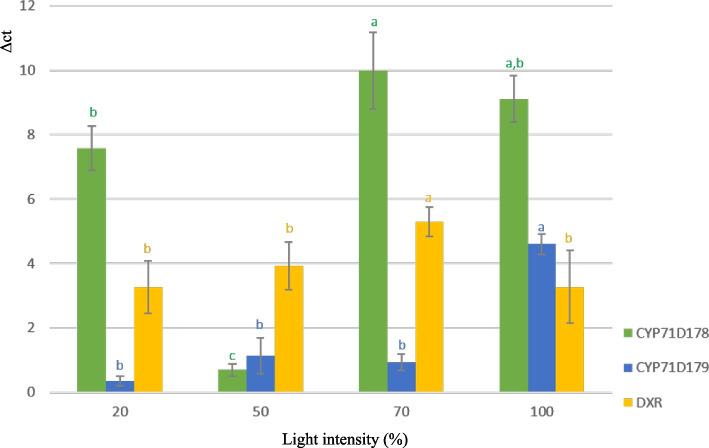
Expression of the MEP pathway key genes under different light intensity treatments (20, 50, 70 and 100%) in *Origanum majorana* L.. The error bars depict the standard deviation, and the use of similar letters indicates that there are no significant differences between the treatments

The expression levels of three genes involved in the MEP were evaluated. Increasing the light intensity from 20 to 50% did not yield any significant difference in *DXR* expression levels. However, at 70% light intensity, *DXR* exhibited its maximum expression level, showing a 62.57% increase compared to the 20% light intensity condition. Afterward, as the light intensity increased, there was a notable decrease of 38.33% in gene expression. *CYP71D178* exhibited the highest expression level at light intensities of 70 and 100%, while demonstrating the lowest at 50%. The expression level of *CYP771D179* in the 100% treatment showed a significant increase, reaching a value 12.52 times higher than that of the 20% light intensity treatment (Fig. 3).

**Fig. 4:**
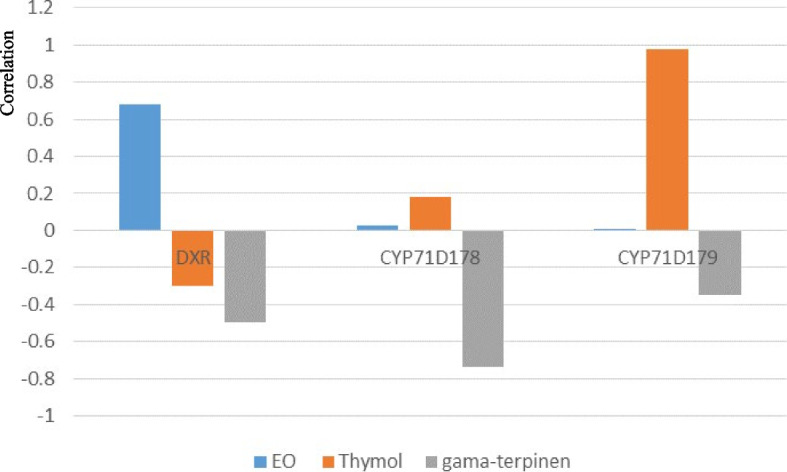
Pearson correlation between the expression of MEP pathway genes and selected components of *Origanum majorana* L. essential oil under varying intensities of natural light treatment

The Pearson correlation test revealed a strong correlation (0.98) between the expression level of the *CYP71D179* gene and the percentage of thymol. Additionally, there was a significant positive correlation (0.68) observed between the expression level of the *DXR* gene and the percentage of essential oil. Conversely, a negative and significant correlation (-0.74) was found between the expression level of the *CYP71D178* gene and the percentage of γ-terpinene (Fig. 4).

## Discussion

Light intensity plays a significant role in regulating both primary and secondary metabolisms, which have an important role in adapting to changing environmental conditions. Effective metabolic adjustments are essential for optimizing plant growth and development while minimizing damage from unfavorable environmental factors. The influence of light on metabolic pathways is mediated by various photoreceptors and the redox system, which provides an adequate supply of reductants and oxidants for biochemical reactions. Furthermore, the photosynthetic and mitochondrial electron transport chains are crucial to this adaptation process, serving as primary sources of reactive oxygen species (ROS) that impact the redox environment within cells and tissues. Variations in the redox environment of subcellular compartments can significantly affect metabolic processes [28]. In this study, increasing light intensity led to elevated levels of primary metabolites, including total sugar and protein content, as well as an increase in plant dry weight (Table 2). When the light intensity is low, plants can increase their chlorophyll levels. This reaction allows them to reduce the light compensation and saturation points, leading to a more efficient absorption of light and an increased rate of photosynthesis. However, prolonged periods of low light intensity can hinder the accumulation of essential compounds and net photosynthetic products necessary for normal growth. As a result, the biomass of plants can only be increased when exposed to full light conditions [29–31]. Previous research has shown that full sunlight conditions significantly elevate sugar content and specific leaf mass in 11 different Lamiaceae species compared to shaded environments. However, no significant variation in soluble protein content was observed across different light conditions [32].

In this study on heightened light intensity, the concentration of H_2_O_2_ in plant cells increases. At the same time, the MDA content rises under full light conditions, indicating membrane degradation (Fig. 1). Elevated MDA levels have been linked to excessive production of ROS, which signifies oxidative damage [33, 34]. Exposure to various environmental stresses can induce oxidative stress in plants, leading to diverse effects [35]. Under these conditions, plants increase the production of reactive oxygen species (ROS). The main sources of H_2_O_2_ generation within plant cells include electron transport chain reactions in chloroplasts and mitochondria, peroxidase enzymes, Nicotinamide adenine dinucleotide phosphate (NADPH) oxidase, peroxisomal oxidases, and other apoplastic oxidases. Accumulation of ROS emerges as a central response in plant leaves under light stress conditions [34–37].

This study discovered that POD and PPO enzymes exhibit maximum activity under low light conditions. In contrast, CAT and SOD enzymes display peak activity under full light conditions. A previous study indicated that when *Festuca arundinacea* was transferred from relatively low-light conditions to high-light conditions, there was an increase in SOD, CAT, and ascorbate peroxidase (APX) activities [38]. These findings underscore the complex regulatory functions of antioxidant enzymes in response to different light conditions, as well as the interplay between light and enzymatic activities in plants. SODs play a crucial role in protecting cells against oxidative damage and are found in all cells. Their main function is to convert or dismutase harmful superoxide (O_2_^−^) radicals into hydrogen peroxide (H_2_O_2_) and molecular oxygen (O_2_) [39]. In plants, SODs are distributed in chloroplasts, cytosol, and mitochondria. CATs, a specific group of heme-containing enzymes, break down hydrogen peroxide into water and oxygen. The enzyme remains inactive when the concentration of H_2_O_2_ is low. At elevated concentrations of H_2_O_2_, this compound acts as a reducing agent, activating the enzyme and resulting in the release of water and oxygen [40]. As a result, an increase in catalase activity in plants is observed parallel to the increase in H_2_O_2_ levels. PODs represent another category of heme-containing proteins that exhibit substantial structural diversity. Their primary function is to oxidize aromatic electron donors using H_2_O_2_ as a substrate [41]. In addition to their essential roles in various biosynthetic pathways, PODs are integral components of defense mechanisms under stressful conditions [42].

This study found that the highest total phenol content occurs at light intensities of 20 and 100%, whereas the lowest level is observed at a light intensity of 50%. On the other hand, the quantity of anthocyanins, which act as antioxidants, peaks at 70% light intensity (Table 3). Medicinal plants can generally be classified into three groups based on their light intensity requirements: heliophytes, sciophytes, and intermediate plants. Heliophytes tend to produce higher levels of secondary metabolites in areas with high light intensity. At the same time, sciophytes thrive in low light intensity conditions and produce more secondary metabolites. Intermediate plants fall somewhere in between these two extremes. When aiming to produce secondary metabolites in medicinal plants, it is crucial to consider concentration and yield as key parameters [43]. In plants, the production of phenolic compounds increases in response to abiotic stress [44]. Phenolic compounds exhibit their antioxidant effectiveness through their high reactivity as providers of hydrogen or electrons. They also help stabilize and disperse unpaired electrons, bind with transition metal ions, and influence both peroxidation kinetics and the scavenging of hydrogen peroxide [45]. In *Mentha spicata*, varying levels of abiotic stress result in increased concentrations of anthocyanin, carotenoids, and total phenolic compounds, which act as a protective response against potential leaf tissue damage [34].

In this study, the level of essential oil content increased until the light intensity reached 70%. It significantly degraded and decreased under full light. Simultaneously, as the light intensity increased, the amounts of compounds such as thymol and trans-sabinene hydrate also increased. On the other hand, compounds such as γ-terpinen, borneol, and bicyclogermacrene were reduced at increasing light intensity (Table 4). The production of essential oil content is influenced by various chemical and environmental factors that collectively affect their susceptibility to oxidation and govern the progression of these reactions [46]. Therefore, it is crucial to carefully consider extrinsic factors such as temperature, light, and atmospheric oxygen availability. Furthermore, the stability of essential oil content is inherently linked to their composition, molecular structures, and the presence of impurities [47]. Importantly, ultraviolet (UV) and visible (Vis) light have been identified as agents that accelerate autoxidation processes by initiating hydrogen atom abstraction, ultimately leading to the formation of alkyl radicals [48, 49]. In a study by Lalevic et al., mint plants grown in shaded conditions produced approximately 44% less essential oil than those grown without shade. Additionally, applying shade treatment led to a decrease in thymol content from 1.4 to 0.3% [10]. A study by Misharina and Polshkov (2005) found that laurel and fennel oils undergo similar compositional changes when stored in the absence of light compared to when they are stored under light exposure. These changes include a decrease in eugenyl acetate, estragol, trans-anethole, and various monoterpenes, along with an increase in *p*-cymene, eugenol, and anisaldehyde. The study also showed that light exposure speeds up the degradation of monoterpenes [50].

However, studies have reported the phytochemical responses of different plant species to varying intensities of light radiation. In this study, we observed the effects of different light intensities on the essential oil content and compounds (Table 4). A study found that thyme oil remained relatively unchanged, while rosemary oil showed increased susceptibility to simulated daylight, resulting in significant changes in its chemical composition. These changes were characterized by an increase in *p*-cymene, camphor, and caryophyllene oxide, along with the degradation of *β*-caryophyllene and monoterpenes such as *β*-myrcene, *α*-terpinene, and *α*-phellandrene [48]. PCA analysis was conducted on the essential oil compounds. The first two components accounted for 100% of the observed variations. Thymol, linalool, and trans-sabinen hydrate were the primary contributors to the first component, while bornyl acetate, bicyclogermacrene, and EO played a significant role in forming the second component.

Higher plants use two pathways, MEP or mevalonate (MVA), for synthesizing isopentenyl diphosphate (IPP)/ dimethylallyl diphosphate (DMAPP), which serve as precursors for monoterpenes. The *DXR* gene is upstream of the MEP pathway [51, 52]. The strong and positive correlation between the expression level of this gene and the percentage of essential oil expression emphasizes its pivotal role in the synthesis of essential oil compounds in the OM plant. CYP71D178 and CYP71D179 can hydroxylate γ-terpene to produce the valuable monoterpene thymol [53]. In OM, there is a highly significant correlation between the expression level of *CYP71D179* and thymol, confirming the impact of this gene on thymol production. On the other hand, the presence of both negative and high correlations between *CYP71D178* and the percentage of *γ*-terpene suggests that an increase in the expression of this gene leads to greater hydroxylation of *γ*-terpinene.

The alteration in light intensity impacts various mechanisms within the plant, ultimately leading to fluctuations in the levels of secondary metabolites, such as terpenes. A reduction in light intensity leads to a decrease in glandular trichomes, the primary source of essential oil production in the plant. In addition to the MEP and MVA pathway genes, other modules are activated or deactivated in response to light signals, influencing terpenes production. For instance, in Arabidopsis plant, the hypocotyl 5 (HY5) and photomorphogenic 1-elongated hypocotyl (PIFs) directly regulate the light-modulated expression of the *DXS* and *DXR* genes, which encode the key enzymes that control flux in the MEP pathway [54, 55]. U*pkhmgr* and U*pkdxs* promoters, located upstream of the genes involved in the photosynthesis of secondary metabolites, respond to changes in light conditions. This response is mediated by light-regulated motifs. For instance, GCCAC boxes have been frequently observed in the promoters of phytochromes, which are influenced by light. Phytochrome is initially produced in the cytosol and subsequently translocates to the nucleus, where it modulates gene expression through interaction with transcription factors [56]. Another factor that affects the production of secondary metabolites in plants is "over flow metabolism." As light intensity increases, the rate of photosynthesis also rises, leading to carbon production that exceeds the carbon demand associated with plant growth. The excess carbon is subsequently directed towards the biosynthesis of secondary metabolites [57, 58].

## Conclusion

The results indicated that an increase in light intensity correlated with elevated levels of metabolites, such as sugars and proteins, in the shoots, ultimately contributing to an increase in the dry weight of the OM plant. The increase in these metabolites was probably due to the increase in the rate of photosynthesis caused by the increase in light availability. The increase of H_2_O_2_ caused by the increase in light intensity led to the accumulation of MDA in the tissues. In addition, variations in light intensity influenced the activity of antioxidant enzymes, thereby enabling the plant to adapt to oxidative stress. At the same time as the light intensity increased to 70%, essential oil increased significantly, but it decreased again at higher light levels.

Despite the increase in the amount of dry matter in full light (100% intensity), the amount of essential oil decreases in this light intensity. Farmers operating in areas with an average light intensity of 1700 μmol/m^2^ per second are advised to utilize shades that permit the transmission of 70% of light to cultivate sweet marjoram in open fields during the final growth stages. This approach optimizes biomass yield and enhances essential oil production. The specific duration of shade effectiveness presents a potential area for future research. Also, people who grow this plant in the greenhouse should reduce the light intensity in the final stages of growth in order to extract more essential oils and reduce energy consumption.

## Supplementary Information

Supplementary Material 1.

## Data Availability

No datasets were generated or analysed during the current study.
