# Supplementary material for: Varying levels of natural light intensity affect the phyto-biochemical compounds, antioxidant indices and genes involved in the monoterpene biosynthetic pathway of Origanum majorana L
Source: BMC Plant Biol. 2024 Oct 28;24:1018. doi: 10.1186/s12870-024-05739-5 (PMC11514805; doi:10.1186/s12870-024-05739-5)
Supplement: Supplementary file 1 — Supplementary Material 1. [file 12870_2024_5739_MOESM1_ESM.docx]

| **Table S1** Analysis of variance of primary metabolite and redox index under varying light intensity in *Origanum majorana* L. | | | | | |
| --- | --- | --- | --- | --- | --- |
| Mean Squares | | | | | |
| Source of variation | Degree of freedom | Protein | Sugar content | H_2_O_2_ | MDA |
| Block | 2 | 16.71 | 0.00006 | 0.00003 | 0.0012 |
| Light intensity | 3 | 3781.9 | 0.00031 | 0.202 | 0.213 |
| Error | 6 | 1.29 | 0.00004 | 0.00002 | 0.0012 |
|  |  | *** | * | *** | *** |
| *** *p*≤ 0.001, **p*≤0.05 | | | | | |

| **Table S2** Analysis of variance of plant anti-oxidant under varying light intensity in *Origanum majorana* L. | | | | | | | | |
| --- | --- | --- | --- | --- | --- | --- | --- | --- |
| Mean Squares | | | | | |  |  |  |
| Source of variation | Degree of freedom | PPO | POD | SOD | CAT | TPC | TFC | Anthocyanin |
| Block | 2 | 12.25 | 17.15 | 0.000004 | 17.96 | 0.005 | 2.1 | 0.00011 |
| Light intensity | 3 | 369.46 | 1179.11 | 0.00015 | 26.13 | 0.323 | 1.169 | 0.00025 |
| Error | 6 | 8.11 | 36.05 | 0.000003 | 189.04 | 0.002 | 1.17 | 0.00003 |
|  |  | *** | ** | *** | * | *** | ns | * |
| *** *p*≤ 0.001, ** *p* ≤0.01, **p*≤0.05, ^ns^ non-significant. | | | | | | | | |

| **Table S3** Analysis of variance of essential oil compounds in *Origanum majorana* L. under light intensity changes | | | | | | | | | | | | | | | | | | |  |
| --- | --- | --- | --- | --- | --- | --- | --- | --- | --- | --- | --- | --- | --- | --- | --- | --- | --- | --- | --- |
| Source of variation | Degree of freedom | | EO | Sabinene | Myrcene | α-Terpinene | *p*-Cymene | Limonene | *b*-Phellandrene | γ-Terpinene | Linalool | trans- Sabinenehydrate | Borneol | Terpinen4ol | α-Terpineol | Bornylacetate | Thymol | Caryophyllene | Bicyclogermacrene |
| Block | | 2 | 0.16 | 0.00002 | 0.012 | 0.0016 | 0.00002 | 0.260 | 0.00022 | 0.011 | 0.0002 | 0.54 | 0.00002 | 0.002 | 0.00002 | 0.0001 | 0.005 | 0.004 | 0.0002 |
| Light intensity | | 3 | 146.69 | 1.61 | 77.31 | 0.059 | 0.067 | 0.479 | 0.177 | 5.69 | 2.86 | 38.78 | 14.68 | 1.022 | 3.28 | 0.053 | 4.87 | 13.79 | 0.12 |
| Error | | 6 | 0.324 | 0.00022 | 0.0004 | 0.0081 | 0.00002 | 0.25 | 0.00002 | 0.002 | 0.0006 | 0.027 | 0.002 | 0.006 | 0.00002 | 0 | 0.0016 | 0.006 | 0.00002 |
|  | |  | * | ** | ** | ns | * | ns | * | * | * | * | * | ns | ** | *** | * | * | * |
| *** *p*≤ 0.001, ** *p* ≤0.01, **p*≤0.05, ^ns^ non-significant. | | | | | | | | | | | | | | | | | | | |

| **Table S4** Component matrix of PCA analysis of essential oil compounds in *Origanum majoraana* L. | | |
| --- | --- | --- |
|  | Component | |
| Essential oil compounds | 1 | 2 |
| Sabinene | .988 | .154 |
| Myrcene | -.664 | .748 |
| *α*-Terpinene | -.884 | .467 |
| *p*-Cymene | .949 | .316 |
| Limonene | .982 | .189 |
| *b*-Phellandrene | .942 | .335 |
| γ-Terpinene | -.972 | -.236 |
| Linalool | .994 | .112 |
| trans-Sabinene hydrate | .993 | .117 |
| Borneol | -.690 | -.724 |
| Terpinen4ol | .977 | -.212 |
| *α*-Terpineol | .988 | .157 |
| Bornylacetate | .382 | .924 |
| Thymol | .995 | -.099 |
| Caryophyllene | -.905 | .425 |
| Bicyclogermacrene | -.562 | .827 |
| EO | .386 | -.922 |
| Mono-terpenes | .687 | .727 |
| Sesqui-terpenes | -.744 | .668 |
| Extraction Method: Principal Component Analysis | | |
